# The Role of Serum Biomarkers for the Differential Diagnosis and Prognostic Assessment of Myocardial Infarction with Non-Obstructive Coronary Arteries: A Narrative Review

**DOI:** 10.3390/jcm15072593

**Published:** 2026-03-28

**Authors:** Matteo Orlandi, Ruggero Mazzotta, Niccolò Ciardetti, Giorgia Panichella, Manuel Garofalo, Lucrezia Biagiotti, Maria Federica Crociani, Samuele Salvi, Carlo Di Mario, Francesco Meucci, Alessio Mattesini

**Affiliations:** 1 Department of Experimental and Clinical Medicine, University of Florence, 50134 Florence, Italy; matteo.orlandi@unifi.it (M.O.); ruggero.mazzotta@unifi.it (R.M.); manuel.garofalo@unifi.it (M.G.); lucrezia.biagiotti@unifi.it (L.B.); mariafederica.crociani@unifi.it (M.F.C.); samuele.salvi@unifi.it (S.S.); carlo.dimario@unifi.it (C.D.M.); 2 Division of Structural Interventional Cardiology, Cardiothoracovascular Department, Careggi University Hospital, 50134 Florence, Italy; ciardettin@aou-careggi.toscana.it (N.C.); meuccif@aou-careggi.toscana.it (F.M.); mattesinia@aou-careggi.toscana.it (A.M.)

**Keywords:** MINOCA, serum biomarkers, acute myocardial injury, differential diagnosis, inflammation, precision medicine, Takotsubo syndrome, myocarditis

## Abstract

Myocardial infarction with non-obstructive coronary arteries (MINOCA) represents a heterogeneous clinical entity encompassing multiple ischemic mechanisms, including atherosclerotic plaque disruption, coronary artery spasm, coronary microvascular dysfunction, coronary embolism, and spontaneous coronary artery dissection. Despite the absence of obstructive coronary disease, patients with MINOCA remain at substantial risk of adverse cardiovascular outcomes, underscoring the need for accurate early diagnosis and effective risk stratification. In this context, accumulating evidence indicates that circulating serum biomarkers may provide additional pathophysiological and prognostic insights in patients with a working diagnosis of MINOCA. Moreover, distinct biomarker profiles may help support the differential diagnostic evaluation between MINOCA and other causes of acute myocardial injury, such as myocardial infarction with obstructive coronary arteries, myocarditis, and Takotsubo syndrome. This narrative review summarizes current evidence on serum biomarkers in MINOCA, highlights their potential role in guiding tailored diagnostic strategies, and discusses future perspectives toward biomarker-driven precision medicine in patients presenting with acute myocardial injury.

## 1. Introduction

Myocardial infarction with non-obstructive coronary arteries (MINOCA) represents a clinical syndrome characterized by evidence of acute myocardial infarction (AMI), as defined by the Fourth Universal Definition of Myocardial Infarction, in the absence of ≥50% coronary stenosis on angiography. As such, MINOCA should be considered a working diagnosis that requires further diagnostic evaluation to determine the underlying mechanism and exclude alternative causes of myocardial injury [1]. MINOCA accounts for approximately 5–15% of all AMI, with a higher prevalence among women and younger individuals [2,3,4]. Despite often being initially perceived as “less severe” than myocardial infarction with obstructive coronary artery disease (MI-CAD), MINOCA is now recognized as a condition with a substantial risk of recurrent adverse cardiovascular (CV) events [5,6].

Pathophysiological substrates of MINOCA are heterogeneous and include atherosclerotic plaque disruption, coronary artery spasm (CAS), coronary microvascular dysfunction (CMD), coronary embolism, and spontaneous coronary artery dissection (SCAD). Non-ischemic disorders such as acute myocarditis and Takotsubo syndrome (TTS) may also manifest with chest pain, electrocardiogram (ECG) alterations, and elevation of cardiac injury biomarkers, mimicking AMI at first presentation [7,8]. These conditions must therefore be carefully excluded before confirming a diagnosis of MINOCA. In addition, clinical scenarios associated with oxygen supply–demand imbalance leading to type-2 myocardial infarction should also be considered and excluded during the diagnostic work-up [7,8].

According to contemporary European and American clinical practice guidelines, patients with a working diagnosis of MINOCA should undergo a structured diagnostic approach integrating clinical assessment and coronary angiography, with intracoronary imaging and functional coronary testing recommended in selected patients. Cardiac magnetic resonance (CMR) is recommended when the diagnosis remains unclear after coronary angiography in order to identify the underlying mechanism of myocardial injury [9,10]. CMR offers excellent tissue characterization and therefore plays a central role in the diagnostic evaluation of patients with suspected MINOCA [11]. Yet, its availability, high cost, limited patient compliance, and contraindications (e.g., arrhythmias, implants, claustrophobia) restrict its universal application in the acute setting, with a diagnostic yield that falls after 14 days from presentation [12].

A biomarker is defined as a characteristic that is objectively measured and evaluated as an indicator of normal biological processes, pathogenic processes, or pharmacologic responses to a therapeutic intervention [13]. In CV medicine, serum biomarkers reflect underlying molecular and cellular pathways involved in myocardial injury, inflammation, and repair, and can therefore aid in diagnosis, risk stratification, and therapeutic guidance [14]. Depending on their clinical application, biomarkers may be broadly categorized as diagnostic, prognostic, predictive, or mechanistic, each with distinct evidentiary requirements. Recent research has increasingly turned to serum biomarkers as key adjuncts in the assessment of MINOCA. These biomarkers offer the promise of earlier risk stratification, mechanistic insight, and potentially lower-cost, widely available alternatives to imaging. Understanding their specific pathophysiological implications may allow clinicians to tailor management strategies to the underlying cause of MINOCA, bridging the current gap between diagnosis and targeted therapy.

This review provides a comprehensive and mechanistically driven overview of current and emerging serum biomarkers in MINOCA, critically analyzing their potential role across diagnostic and prognostic frameworks, including differentiation of MINOCA from MI-CAD, acute myocarditis and TTS.

**Figure 1:**
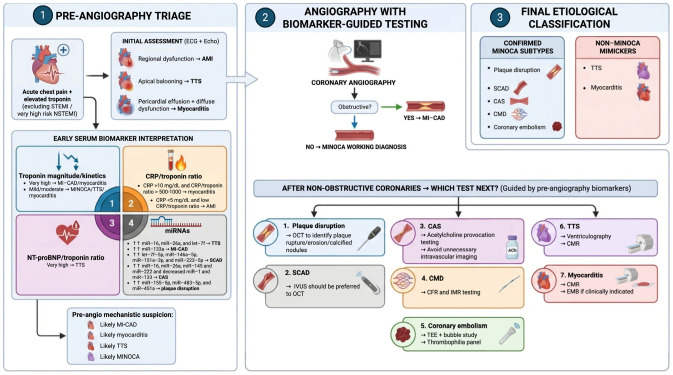
The figure illustrates a three-step pathway. (**1**) Pre-angiography triage combines clinical presentation, ECG/echocardiography findings, and early serum biomarker interpretation. Troponin magnitude and kinetics, CRP/troponin ratio, NT-proBNP/troponin ratio, and selected circulating miRNAs provide early mechanistic clues, allowing preliminary differentiation among MINOCA, MI-CAD, myocarditis, and TTS. (**2**) Coronary angiography with biomarker-guided testing establishes the presence or absence of obstructive coronary disease and directs subsequent diagnostic testing in patients with non-obstructive coronaries. Depending on the suspected mechanism, targeted investigations include OCT for plaque disruption, IVUS for suspected SCAD, acetylcholine provocation testing for CAS, CFR and IMR assessment for CMD, TEE and thrombophilia testing for embolic MI, and CMR for suspected myocarditis or TTS. (**3**) Final etiological classification distinguishes confirmed MINOCA subtypes from non-MINOCA mimickers. Abbreviations: AMI, acute myocardial infarction; CAS, coronary artery spasm; CFR, coronary flow reserve; CMR, cardiac magnetic resonance; CMD, coronary microvascular dysfunction; CRP, C-reactive protein; ECG, electrocardiogram; EMB, endomyocardial biopsy; IMR, index of microcirculatory resistance; IVUS, intravascular ultrasound; MI-CAD, myocardial infarction with obstructive coronary artery disease; miRNAs, microRNAs; NSTEMI, non-ST-elevation myocardial infarction; NT-proBNP, N-terminal pro-B-type natriuretic peptide; OCT, optical coherence tomography; SCAD, spontaneous coronary artery dissection; STEMI, ST-elevation myocardial infarction; TEE, transesophageal echocardiography; TTS, Takotsubo syndrome.

The schematic representation for biomarker-guided evaluation of acute myocardial injury is illustrated in Figure 1. The figure is intended to provide a conceptual overview of potential diagnostic pathways rather than a prescriptive or guideline-endorsed algorithm.

## 2. Materials and Methods

This narrative review was conducted through a structured search of the biomedical literature. PubMed/MEDLINE and Scopus databases were searched for relevant articles published up to January 2026. The search strategy included combinations of the following keywords: “MINOCA”, “myocardial infarction with non-obstructive coronary arteries”, “biomarkers”, “serum biomarkers”, “troponin”, “C-reactive protein”, “natriuretic peptides”, “microRNAs”, “Takotsubo syndrome”, and “myocarditis”. Additional relevant publications were identified through manual screening of reference lists from selected articles and review papers. Original research articles, observational studies, and relevant clinical reviews addressing serum biomarkers in the diagnostic or prognostic evaluation of MINOCA were considered. Given the narrative nature of the review, study selection was based on relevance to the topic and on the methodological quality and clinical significance of the available evidence. Preference was given to studies with larger cohorts, multicenter registries, and guideline or consensus documents from major cardiovascular societies.

During the preparation of this manuscript, the author used ChatGPT (GPT-5.3; OpenAI, San Francisco, CA, USA) solely to assist with English language editing and to improve clarity and readability. All scientific content, data interpretation, statistical analyses, and conclusions were entirely conducted and verified by the author.

## 3. Pathophysiology of MINOCA

Atherosclerotic plaque disruption, including plaque rupture and erosion, or destabilization of calcified nodules, is a frequent cause of MINOCA. Intravascular imaging studies using intravascular ultrasound (IVUS) have reported plaque disruption in approximately 40% of cases [15,16]. Such lesions may not cause flow-limiting stenosis but are sufficient to trigger thrombosis and distal embolization, leading to myocardial necrosis.

CAS contributes significantly to MINOCA pathophysiology. Spasm may affect both epicardial and microvascular coronary arteries and is associated with vascular smooth muscle hyperreactivity, endothelial dysfunction, and exposure to vasospastic substances [17]. In patients presenting with MINOCA and suspected coronary vasomotor abnormalities, a provocative test was positive in 46.2% of patients [18].

CMD is another relevant mechanism, particularly in women, that may result from structural microvascular remodeling, endothelial dysfunction, or increased sympathetic drive, impairing coronary blood flow despite normal epicardial arteries [19,20].

Coronary embolism is a less common cause of MINOCA but it should be considered, especially in younger patients or those with predisposing conditions, such as in the case of inherited or acquired thrombophilia (e.g., malignancies, antiphospholipid syndrome, myeloproliferative diseases) [2,21], atrial fibrillation, infective endocarditis, prosthetic heart valves, and a patent foramen ovale predisposing to paradoxical embolism [7,8,22].

SCAD is a non-atherosclerotic cause of MINOCA particularly affecting younger women without traditional CV risk factors. SCAD consist in the development of an intimal tear, with or without intramural hematoma, determining a dynamic obstruction of coronary flow. It is often associated with connective tissue disorders and could develop in the presence of structural abnormalities in the arterial wall, such as dysregulation of the transforming growth factor-β (TGF-β) signaling pathway, fibrillin-1 gene mutations, and extracellular matrix protein alterations [23].

## 4. Acute Myocarditis and Takotsubo Syndrome: Mimickers of MINOCA

In a retrospective cohort study of patients referred for CMR because of a working diagnosis of MINOCA, up to 78% of cases were reclassified after imaging, most commonly as myocarditis (24%) or TTS (13%). It should be noted that these findings derive from patients selected for CMR evaluation, which may influence the observed diagnostic yield [24]. These results highlight the substantial overlap in clinical presentation among ischemic and non-ischemic causes of myocardial injury. Non-ischemic conditions such as acute myocarditis and TTS often present with chest pain, troponin elevation, and ECG changes, mimicking AMI and leading to an initial diagnosis of MINOCA.

Myocarditis is characterized by myocardial inflammation typically of viral or autoimmune origin. Clinical presentation often mirrors AMI; however, the pathophysiologic mechanism is direct myocyte injury due to inflammation rather than ischemia. CMR is central for diagnosis, demonstrating myocardial edema and non-ischemic late gadolinium enhancement pattern [11]. Treatment is mainly supportive and directed at managing inflammation and ventricular dysfunction [25].

TTS is an acute stress-induced condition, most common in postmenopausal women, and characterized by transient regional wall motion abnormalities extending beyond a single coronary territory. The pathophysiology is thought to involve catecholamine-mediated myocardial stunning. CMR reveals edema without ischemic scar and therefore helps differentiate TTS from AMI [26].

Although myocarditis and TTS fulfill the clinical definition of AMI at presentation, their mechanisms are non-ischemic. This distinction has therapeutic implications, as standard secondary prevention therapies for ischemic myocardial injury may not be appropriate. For this reason, experts suggest their early exclusion through multimodality imaging, particularly CMR, and regard them as separate entities that mimic MINOCA rather than being part of its spectrum [7,8].

## 5. Integrating Serum Biomarkers into the Diagnostic Pathway of Acute Myocardial Injury

The differential diagnosis between MINOCA and other causes of acute myocardial injury (namely MI-CAD, acute myocarditis, and TTS) is a crucial step in the early evaluation of patients presenting with acute chest pain. These entities often share similar clinical presentations, ECG patterns, and troponin elevations, but differ substantially in underlying mechanisms, prognosis, and treatment strategies. In this context, serum biomarkers, interpreted alongside clinical data and imaging, may help refine the working diagnosis while patients are still in the acute phase. Below, the available evidence on serum biomarkers is organized according to their role in distinguishing MINOCA from MI-CAD, MINOCA from myocarditis, and MINOCA from TTS, while preserving the complex and often overlapping nature of these conditions.

A comparative overview of biomarker behavior across MI-CAD, MINOCA, TTS, and myocarditis is presented in Table 1.

### 5.1. Serum Biomarkers to Differentiate MINOCA from MI-CAD

#### 5.1.1. Myocardial Injury Biomarkers

Cardiac-specific troponins I and T remain the central biomarkers for diagnosing AMI, including MINOCA. Several studies have consistently shown that patients with MINOCA generally exhibit lower peak troponin levels compared with those with MI-CAD, suggesting a smaller extent of myocardial necrosis on average [27,28,29,30,31]. In the multicenter VIRGO (Variation in Recovery: Role of Gender on Outcomes of Young AMI Patients) study, which prospectively enrolled young AMI patients (18–55 years) with a 2:1 female-to-male ratio, an angiography-based classification was used: patients were categorized as MI-CAD if revascularization was performed or angiography showed plaque ≥ 50%, and as MINOCA if coronary stenosis was <50% or a nonplaque mechanism was identified (e.g., CAS relieved by intracoronary nitroglycerin, SCAD, or coronary embolism) [58]. Across this spectrum, peak troponin values appeared higher in patients with MI-CAD than in those with MINOCA [58]. However, this pattern is not universal. In a case–control study, Daniel et al. compared 100 patients with AMI and angiographically normal or near-normal coronary arteries to 100 patients with AMI and obstructive CAD and 100 healthy controls. Relative troponin (I or T) levels were similar between the two AMI groups, suggesting that troponin magnitude alone may not reliably differentiate MINOCA from MI-CAD in all settings [43]. It is important to note that TTS cases were included among the “normal coronaries” group, while myocarditis was excluded by CMR. This methodological aspect may have substantially influenced the observed biomarker patterns and should be considered when interpreting these findings. Among elderly patients, troponin appears more informative. In a single-center registry of 324 patients ≥ 70 years admitted with non-ST-elevation myocardial infarction (NSTEMI), 21.9% were ultimately classified as MINOCA [32]. These patients showed lower peaks of high-sensitivity cardiac troponin T (hs-cTnT) compared with those with MI-CAD. On multivariate analysis, several variables were independently associated with a MINOCA diagnosis: female sex, absence of prior AMI, chest pain at rest, absence of heart failure (HF) signs (Killip class ≥ 2), absence of ST-segment depression, left bundle branch block, pacemaker rhythm, and lower troponin peaks. The composite model yielded good discrimination [area under the curve (AUC) = 0.83], although internal validation methods were not reported [32]. Similarly, in the large ARIAM-SEMICYUC (Analysis of Delay in AMI-Spanish Society of Intensive Care Medicine and Coronary Unit) registry, which included 752 MINOCA and 9241 MI-CAD patients, fewer MINOCA patients exhibited troponin elevations greater than nine times the local reference limit compared with MI-CAD, confirming that extreme troponin elevations are more typical of obstructive infarction [33].

Creatine kinase-myocardial band (CK-MB), historically central to AMI diagnosis, adds little beyond high-sensitivity troponin in contemporary practice. Nonetheless, its behavior parallels that of troponin in this context: in at least one study, CK-MB values were lower in MINOCA than in MI-CAD, mirroring the more limited extent of necrosis in the former [28].

Overall, cardiac injury biomarkers are highly sensitive but not mechanism specific. Their clinical interpretability may improve when considered alongside imaging modalities such as early CMR, which provides tissue characterization and can help clarify the underlying etiology of myocardial injury [12,59]. Pathik et al. investigated 125 patients with suspected MINOCA undergoing early CMR, finding that while troponin was universally elevated, CMR can identify a definitive etiology in up to 60–70% of cases and reclassifies the initial clinical diagnosis in more than two-thirds of patients [11].

#### 5.1.2. Inflammation Biomarkers

Systemic inflammation is recognized as a critical factor not only in the acute phase of AMI but also in the progression of atherosclerotic disease and plaque instability [60]. The growing attention of inflammatory biomarkers in CV research reflects their potential to improve pathophysiological understanding and patient risk stratification [61]. Furthermore, inflammation has emerged as a promising therapeutic target, as anti-inflammatory treatments have been shown to reduce CV events in patients with AMI and chronic CAD [62]. Inflammatory activation is implicated in several mechanisms of MINOCA: systemic inflammation and pericoronary inflammation may actually trigger CAS, CMD, and destabilization of angiographically non-significant atherosclerotic plaques [63,64,65].

Several studies suggest that the inflammatory milieu differs between MINOCA and MI-CAD, although findings are not entirely consistent [29,34,43,44,45]. In the study by Daniel et al., C-reactive protein (CRP) > 5 mg/L was present in 16% of MI-CAD patients, 13% of patients with AMI and no or minimal atheromatosis (a group including MINOCA and TTS), and 5% of healthy controls. While CRP did not significantly differ between MI-CAD and non-obstructive AMI, patients with AMI and non-obstructive coronaries had higher CRP than healthy controls, confirming that systemic inflammation accompanies myocardial injury regardless of the angiographic pattern [43]. A post hoc analysis of the PLATO (Platelet Inhibition and Patient Outcomes) trial provided additional insight. Patients with MINOCA had lower baseline hs-cTnT, higher high-sensitivity C-reactive protein (hs-CRP), and growth differentiation factor 15 (GDF-15), and similar N-terminal pro-B-type natriuretic peptide (NT-proBNP) concentrations compared with MI-CAD. At one month, hs-CRP and hs-cTnT levels were lower in MINOCA, while GDF-15 remained comparable between groups [34]. GDF-15, a stress-responsive cytokine of the TGF-β superfamily, is an established marker of adverse outcomes in AMI and other CV conditions [66]. It is upregulated in ischemic cardiomyocytes and is associated with inflammation, oxidative stress, and hypoxia [66]. In PLATO, GDF-15 was initially higher in MINOCA than in MI-CAD but converged at follow-up, suggesting that it may reflect chronic systemic risk more than acute plaque-related events [34]. Data from the SWEDEHEART registry (9916 MINOCA and 97,970 MI-CAD patients) showed similar median admission CRP in both groups (5.0 [2.0–9.0] mg/dL and 5.0 [2.1–10.0] mg/dL, respectively), indicating that in the acute phase CRP alone is insufficient to distinguish between obstructive and non-obstructive AMI phenotypes [46]. A more refined approach was proposed in a prospective study that developed a biomarker-based index to differentiate MINOCA from MI-CAD prior to angiography [47]. In 111 AMI patients (46 MINOCA, 65 MI-CAD), hs-CRP, interleukin-6 (IL-6), asymmetric dimethylarginine (ADMA), and peak hs-cTnT were measured within 24 h. Hs-CRP and ADMA did not differ significantly between the two groups, whereas MI-CAD patients had higher hs-cTnT (858.0 ng/mL [195.8–2756.5] vs. 95.2 ng/mL [46.0–228.0]; *p* < 0.001) and higher IL-6 (7.4 pg/mL [4.1–15.5] vs. 4.1 pg/mL [2.3–8.3]; *p* = 0.005). Logistic regression confirmed that higher IL-6 and hs-cTnT increased the probability of MI-CAD [odds ratio (OR) 1.58 (95% confidence interval [CI] 1.01–2.46) and OR 2.27 (95% CI 1.61–3.26), respectively], whereas ADMA had an inverse relationship. The combined four-marker index achieved excellent discrimination (AUC = 0.918), outperforming each biomarker alone and offering a promising integrated diagnostic tool. However, these findings derive from a relatively small cohort and should be interpreted with caution, as the model may be susceptible to overfitting and has not yet been externally validated [47]. In a large cohort of AMI patients, CRP, neutrophil-to-lymphocyte ratio (NLR), platelet-to-lymphocyte ratio (PLR), and neutrophil-to-platelet ratio (NPR) were evaluated along with admission glucose and indices of infarct size. In both MI-CAD and MINOCA, admission hyperglycemia was associated with higher NLR, NPR, and PLR, reflecting an enhanced inflammatory state. While admission inflammatory markers were similar between hyperglycemic MI-CAD and MINOCA, white blood cell count (WBCc) and neutrophils at 24 h were significantly higher in MI-CAD [67].

Not all studies have found robust inflammatory differences between MINOCA and MI-CAD. Some earlier reports failed to identify significant CRP or hs-CRP differences between the two entities, possibly because they focused on the acute phase, where myocardial necrosis itself drives CRP elevation [28,48]. To better capture chronic inflammatory activity, Hjort et al. measured 91 biomarkers three months after AMI in 97 MINOCA patients, 97 MI-CAD patients, and 98 controls. Using Lasso regression, eight biomarkers distinguished MINOCA from MI-CAD: P-selectin glycoprotein ligand-1 (PSGL-1), C-X-C motif chemokine ligand 1 (CXCL-1), TNF-related activation-induced cytokine (TRANCE), and pappalysin-1 (PAPPA) were associated with MINOCA, whereas tissue-type plasminogen activator (tPA), B-type natriuretic peptide (BNP), myeloperoxidase (MPO), and interleukin-1 receptor antagonist protein (IL-1Ra) were associated with MI-CAD. Comparing MINOCA with controls, NT-proBNP, renin (REN), nuclear factor-κB essential modulator (NEMO), PAPPA, IL-6, and soluble urokinase plasminogen activator surface receptor (suPAR) were higher in MINOCA, while agouti-related protein (AGRP) was higher in controls. These findings are of interest but should be interpreted cautiously given the relatively small cohort and the exploratory nature of this high-dimensional biomarker analysis. Strikingly, MINOCA patients had lower hs-CRP than MI-CAD, yet similar hs-CRP to controls, despite clear elevation of more specific inflammatory mediators [44]. This suggests that hs-CRP alone may underestimate chronic inflammatory activation in MINOCA, and that multi-marker panels may better capture ongoing vascular and myocardial inflammation. A smaller longitudinal study further supported this concept. Sixteen MINOCA and 21 MI-CAD patients had multiple measurements of inflammatory biomarkers in the early post-infarction phase and at 1 year. On admission, hs-CRP was paradoxically higher in MINOCA but became comparable thereafter and normalized by 1 year. While several chemokines [C-X-C motif chemokine ligand 6 (CXCL-6), LIGHT, C-C motif chemokine ligand 8 (CCL-8), and endocan-1] were similar between groups, MINOCA patients exhibited higher levels and dynamic changes of placental growth factor (PlGF), oncostatin M (OSM), interleukin-20 (IL-20), C-C motif chemokine ligand 15 (CCL-15), C-C motif chemokine ligand 21 (CCL-21), and soluble vascular cell adhesion molecule-1 (sVCAM-1) over time. Notably, in MINOCA, sVCAM-1 and CCL-21 at day 7 predicted atherosclerosis progression at computed tomography angiography, whereas in MI-CAD, CCL-8 and CXCL-6 at day 7 were the main determinants [29]. These findings indicate that the inflammatory signature and its temporal evolution differ between obstructive and non-obstructive AMI and that selected chemokines may be involved in disease progression even when angiography is initially “normal.”

#### 5.1.3. Cardiometabolic Biomarkers

Prior investigations consistently indicate that dyslipidemia is less prevalent among MINOCA patients than MI-CAD patients, suggesting a distinct risk factor profile [2,5]. In the case–control study by Daniel et al., patients with AMI and angiographically normal coronaries (including TTS) had lower triglycerides (TG) and low-density lipoprotein cholesterol (LDL-C), and higher high-density lipoprotein cholesterol (HDL-C) compared with MI-CAD, further supporting less pronounced atherogenic dyslipidemia in the non-obstructive group [43]. An observational study of 114 MINOCA patients and 110 controls added nuance to this picture. MINOCA patients had higher mean total cholesterol (TC), HDL-C, and non-HDL-C, whereas TG, LDL-C, and very low-density lipoprotein cholesterol were slightly lower; none of these differences reached statistical significance. The classic lipoprotein ratios (TC/HDL-C, LDL-C/HDL-C, TG/HDL-C, non-HDL-C/HDL-C), which integrate information on atherogenic and protective lipoproteins, were also lower in MINOCA and remained well below thresholds commonly associated with increased coronary risk. Interestingly, remnant cholesterol (RC) was significantly higher in MINOCA than in those with MI-CAD (21.3 ± 10.6 vs. 13.2 ± 7.7 mg/dL, *p* < 0.0001), although this observation derives from unadjusted analyses. In the same cohort, the monocyte-to-HDL-C ratio was higher (23 vs. 18.5, *p* = 0.05), and PLR tended to be higher (179.8 ± 246.1 vs. 135 ± 64.7, *p* = 0.05), though the latter did not reach statistical significance [56]. These findings point towards a more subtle interplay between dyslipidemia, inflammation, and residual risk in MINOCA compared with classic MI-CAD.

Lipoprotein(a) [Lp(a)], a genetically determined low density lipoprotein-like particle with proatherogenic and prothrombotic properties, has been linked to a higher risk of ischemic CV events in multiple settings [68,69,70,71]. In a multicenter cohort of 1042 AMI patients (76 MINOCA), only 9.2% of MINOCA patients had Lp(a) > 60 mg/dL, compared with 19.8% of MI-CAD. At stepwise logistic regression, elevated Lp(a) levels were more strongly associated with MI-CAD than with MINOCA [OR 0.35 (95% CI 0.14–0.84); *p* = 0.019, supporting the concept that marked Lp(a) elevation is more typical of obstructive atherothrombotic infarction [45].

#### 5.1.4. Biomarkers of Increased Prothrombotic Activity

Coronary thromboembolism represent another major mechanism of MINOCA. In a small comparative study including 16 MINOCA and 21 MI-CAD patients, the authors found increased prothrombotic activity in MINOCA, characterized by greater homocysteine elevation and plasminogen deficiency [31]. However, these findings derive from a very limited cohort and should be interpreted cautiously, as the small sample size and potential referral bias may influence the observed associations. These results also differ from prior studies reporting no differences in thrombophilia markers [2,72], likely reflecting ethnic and methodological variability.

#### 5.1.5. Natriuretic Peptides

Natriuretic peptides complement troponins in distinguishing MINOCA from MI-CAD by reflecting the degree of myocardial stress and dysfunction. In the previously mentioned study by Hjort et al., MINOCA patients displayed higher NT-proBNP than controls but lower BNP than MI-CAD at 3 months, indicating an intermediate degree of myocardial dysfunction: worse than healthy individuals but less severe than classic MI-CAD [44]. In another observational study including MI-CAD, MINOCA, TTS, and healthy controls, NT-proBNP levels at three months were significantly higher in MINOCA and TTS than in healthy controls, but lower than in MI-CAD patients. Intriguingly, patients fulfilling diagnostic criteria for TTS had similar NT-proBNP levels and clinical characteristics to those with MINOCA, reinforcing the diagnostic overlap between these entities and the need for integrated biomarker and imaging-based evaluation [43].

In summary, in the MI-CAD vs. MINOCA comparison, no single serum biomarker provides definitive discrimination. Rather, patterns—such as higher troponin, more pronounced inflammatory activation (IL-6, CRP), higher Lp(a), and distinct multi-marker indices—favor MI-CAD, whereas lower troponin, less overt dyslipidemia, absence of high Lp(a), and different inflammatory signatures may support a MINOCA phenotype, particularly when interpreted alongside CMR and clinical context. However, these patterns remain investigational and are not currently incorporated into guideline-recommended diagnostic algorithms.

### 5.2. Serum Biomarkers in the Differential Diagnosis Between MINOCA and Myocarditis

Acute myocarditis often presents as an ACS mimic, with chest pain, ECG changes, and troponin rise, but without obstructive CAD on angiography. In this setting, inflammatory biomarkers, particularly CRP and its relationship to troponin, are especially valuable. Myocarditis typically features variable troponin elevation (from modest to marked), reflecting the extent and patchiness of myocardial inflammation, and is often accompanied by pronounced CRP elevation and, in some cases, BNP/NT-proBNP elevation indicating myocardial strain [25,40]. One particularly useful concept is the CRP/troponin ratio. A very high CRP relative to troponin strongly favors an inflammatory myocardial process (such as myocarditis) over ischemic necrosis. In a pooled cohort of 556 patients initially labeled as MINOCA, CRP > 10 mg/dL strongly suggested myocarditis and prompted reevaluation with CMR or, when indicated, endomyocardial biopsy (EMB) [51]. Conversely, CRP < 5 mg/dL was associated with imaging findings more consistent with small-vessel obstruction or ischemic MINOCA in a cohort of 135 presumed MINOCA patients [52]. In a large single-center registry including 1898 consecutive patients (1025 STEMI, 518 NSTEMI, and 355 myopericarditis), CRP and troponin were systematically measured at admission, and their ratio (CRP/troponin) was evaluated against the final discharge diagnosis. Median admission CRP/troponin ratios were 84 mg × mL/L × ng in STEMI, 65 mg × mL/L × ng in NSTEMI, and 436 mg × mL/L × ng in myopericarditis, clearly demonstrating a substantial shift towards higher CRP/troponin values in inflammatory myocardial injury. Receiver operating characteristic analyses showed that the admission CRP/troponin ratio had a good discriminatory capacity for myopericarditis versus all AMI (AUC = 0.74), ST-elevation myocardial infarction (STEMI; AUC = 0.73), and NSTEMI (AUC = 0.77). Thresholds were derived from the study data, including a Youden index around 500. At a CRP/troponin ratio > 500, sensitivity and specificity for myopericarditis versus all AMI were 50.0% and 86.9%, respectively; at a ratio > 1000, sensitivity was 39.9% and specificity 94.0%, indicating that very high CRP/troponin values strongly favor an inflammatory rather than ischemic etiology [53].

In this context, CRP is a useful discriminator between ischemic and inflammatory causes of myocardial injury when interpreted in relation to troponin and the clinical picture. Therefore, in patients with non-obstructive coronaries and elevated troponin, marked CRP elevation (especially >10 mg/dL) and a disproportionately high CRP/troponin ratio should raise strong suspicion of myocarditis, prompting urgent CMR and appropriate immunological or infectious work-up, whereas normal or only mildly elevated CRP favors MINOCA or TTS.

### 5.3. Serum Biomarkers in the Differential Diagnosis Between MINOCA and Takotsubo Syndrome

TTS frequently presents as an ACS mimic with chest pain, ST-segment changes, and troponin elevation, and coronary angiography typically shows non-obstructive coronaries [37,38,39]. Differentiating TTS from MINOCA is often challenging in the acute phase. Here, natriuretic peptides, troponin ratios, and circulating microRNAs (miRNAs) provide important diagnostic clues.

TTS is characterized by a disproportionate rise in natriuretic peptides relative to markers of necrosis. Madhavan et al. showed that, compared with STEMI, TTS patients had much higher BNP levels but lower troponin and CK-MB, reflecting severe transient left ventricular dysfunction with relatively limited permanent necrosis. The BNP/peak cardiac troponin T (cTnT) ratio distinguished TTS from STEMI with a sensitivity of 94% and specificity of 100% [41]. In another prospective study of 39 TTS, 48 STEMI, and 34 NSTEMI patients, serial NT-proBNP, cTnT, CK-MB, and myoglobin were measured over the first three days. The investigators evaluated several biomarker ratios to differentiate TTS from AMI. At admission, a NT-proBNP/myoglobin ratio > 3.8 differentiated TTS from STEMI with 89% sensitivity and 90% specificity, while a ratio > 14 differentiated TTS from NSTEMI with 65% sensitivity and 90% specificity. For peak values, a NT-proBNP/cTnT ratio > 2889 distinguished TTS from STEMI (sensitivity 91%, specificity 95%), and >5000 distinguished TTS from NSTEMI (sensitivity 83%, specificity 95%) [42]. These ratios capture the hallmark of TTS: marked hemodynamic and wall-stress activation (high NT-proBNP) disproportionate to the limited necrosis (relatively low troponin and myoglobin); however, it must be considered that the proposed thresholds remain derived from the original study cohorts and have not yet undergone external validation. As outlined above, NT-proBNP levels at 3 months are also higher in both TTS and MINOCA compared with healthy controls but lower than in MI-CAD, and patients fulfilling TTS criteria may resemble MINOCA clinically and biochemically [43]. Therefore, it is the relative pattern (and especially the ratios between NT-proBNP and necrosis markers) rather than absolute values alone that best discriminates TTS from MINOCA.

The role of circulating miRNAs as molecular markers has gained particular interest in TTS, where neurohumoral stress, endothelial dysfunction, and microvascular spasm are central. In one study, broad miRNAs profiling followed by targeted validation in 36 TTS patients, 27 STEMI patients, and 28 healthy controls identified a unique circulating miRNAs signature [57]. miR-16 and miR-26a were significantly upregulated in TTS compared with healthy subjects, and miR-16, miR-26a, and let-7f were higher in TTS than in STEMI. Conversely, the cardiac-specific miRNAs miR-1 and miR-133a were markedly elevated in STEMI compared with healthy controls, and miR-133a was significantly higher in STEMI than TTS. A composite miRNAs panel comprising miR-1, miR-16, miR-26a, and miR-133a distinguished TTS from healthy controls [AUC = 0.835 (95% CI 0.733–0.937)] and from STEMI [AUC = 0.881 (95% CI 0.793–0.968)] with high diagnostic accuracy. In addition, TTS patients showed downregulation of miR-125a-5p, a known negative regulator of endothelin-1 (ET-1), together with significantly increased ET-1 plasma levels [57]. This supports the pathophysiological model in which stress-related miRNAs and ET-1–mediated endothelial dysfunction drive coronary vasoconstriction and transient myocardial stunning.

The combined upregulation of stress- and depression-associated miRNAs (miR-16, miR-26a, let-7f), the ET-1/miR-125a-5p imbalance, and the disproportionate elevation of natriuretic peptides relative to necrosis markers provide a molecular fingerprint that helps separate TTS from MINOCA and from MI-CAD, particularly when CMR and coronary imaging are inconclusive or delayed. However, the clinical translation of circulating miRNAs remains limited by pre-analytical variability, assay heterogeneity, and the lack of standardized measurement protocols, which may affect reproducibility across studies.

## 6. Biomarker-Based Differentiation of MINOCA Subtypes

Differentiating the multiple etiologies of MINOCA represents a core challenge due to overlapping clinical presentations. However, emerging evidence indicates that specific biomarker patterns align with distinct mechanistic subsets of MINOCA, offering potential diagnostic guidance prior to invasive testing.

The key biomarkers reflecting the underlying pathophysiological mechanisms of MINOCA etiologies are presented in Table 2.

Cardiac troponin kinetics differ substantially among the various MINOCA mechanisms. In the VIRGO study, embolic MINOCA demonstrated the highest median peak troponin values expressed as multiples of the upper limit of normal (ULN) (33.1 [17.7–33.7] × ULN), followed by SCAD (12.1 [5.2–35.3] × ULN), whereas CAS and undefined MINOCA exhibited lower peaks (1.5 [0.4–21.4] × ULN and 2.3 [0.8–6.7] × ULN, respectively). Importantly, these etiologic categories were primarily assigned based on angiographic assessment rather than systematic imaging confirmation [5]. These findings support the observation that troponin release correlates with the magnitude and mechanism of ischemic injury.

Inflammation is increasingly recognized as a relevant contributor in several MINOCA etiologies. Higher concentrations of CRP and IL-6 have been reported in patients with CAS and CMD, suggesting a potential association between inflammatory activation and endothelial dysfunction or coronary vasomotor abnormalities [65,73,74,75]. CRP has been proposed to influence endothelial function through reduced nitric oxide bioavailability, while cytokine-mediated pathways have been hypothesized to contribute to coronary vasospasm [65,76]. Increased hs-CRP levels have also been associated with CMD in patients without routine CV risk factors, correlating with ischemic ST-segment alterations [65]. Additionally, serum hs-CRP concentrations are greater in patients with rather than without CAS, supporting a possible relationship with coronary vasomotor dysfunction [77]. IL-6, a marker of atherothrombotic risk, has been found elevated in non-obstructive coronary disease including CMD and CAS, and may have potential prognostic and diagnostic utility in MINOCA [74,78,79]. Elevated NLR, white blood cell count-to-mean platelet volume ratio (WMR), and NPR have been reported in specific subtypes of MINOCA, particularly plaque rupture and CMD, and may reflect the contribution of neutrophil-driven inflammation and platelet activation. However, these findings are primarily associative and their mechanistic significance remains to be clarified [80].

Beyond systemic inflammation, epicardial adipose tissue (EAT) and pericoronary adipose tissue (PCAT) play an emerging mechanistic role. PCAT-derived pro-inflammatory cytokines were independently associated with the occurrence of major adverse cardiovascular events (MACE) in MINOCA patients [81]. Normally releasing anti-inflammatory and vasodilatory mediators such as adrenomedullin, EAT and PCAT may shift toward a pro-inflammatory phenotype under metabolic stress, favoring macrophage M1 polarization, reactive oxygen species production, cytokine release, and local endothelial dysfunction [82]. Perivascular inflammation has been shown to be greater in CAS, supporting the hypothesis that vasospasm may be driven or amplified by local inflammatory activity [63,83].

Other inflammation-related markers, such as cell adhesion molecules, have been associated with CMD and CAS [84,85]. Particular attention has been given to suPAR, which reflects systemic inflammatory activation and endothelial dysfunction. Elevated levels were associated with increased mortality and MACE in MINOCA [73,86,87,88]. Plasminogen activator inhibitor-1 (PAI-1) produced within coronary circulation has been linked to epicardial endothelial dysfunction and may contribute to myocardial ischemia in vasomotor disorders [88]. Serotonin is a mediator of vascular smooth muscle constriction and may serve as a predictive marker of CAS and CMD [89]. Likewise, neuropeptide-Y (NPY), a sympathetic co-transmitter, correlates with increased myocardial injury in CMD and predicts worse prognosis when elevated [90,91]. Renalase elevation has been suggested as a potential screening biomarker for CMD in patients presenting with acute chest pain [92]. Cystatin-C has emerged as a marker of microvascular injury, as elevated levels were independently associated with multivessel CAS severity [93,94]. New proteomic biomarkers including selenium binding protein-1 (SELENBP1) and vinculin (VCL) have been found reduced in CAS and may reflect impaired vascular structural integrity [95]. ET-1, a potent vasoconstrictor, is also implicated in MINOCA. Increased ET-1 concentrations have been observed in MINOCA patients with CAS or CMD, reinforcing the contribution of exaggerated vasoconstrictive drive to transient ischemia in the absence of obstructive CAD [96]. Elevated plasma levels of soluble CD40-ligand (sCD40L), an inflammatory biomarker associated with inflammation and thrombus formation, have been described in patients with AMI due to acute plaque destabilization [97].

D-dimer may support embolic etiology, prompting screening for atrial fibrillation, intracardiac thrombus, valvular prosthesis dysfunction, or non-cardiac embolic sources, although its specificity remains limited [7,8]. Importantly, hereditary thrombophilia has been described in 14–33% of MINOCA patients [2,21,72], including protein C and S deficiencies and factor V Leiden mutation. However, this prevalence varies widely depending on patient selection and the extent of thrombophilia testing, as screening strategies and diagnostic panels differ across studies. Other associations include hyperhomocysteinemia and elevated von Willebrand factor [98,99].

SCAD appears mechanistically linked to abnormalities in the TGF-β pathway, vascular smooth muscle cytoskeletal integrity, eosinophilic activation, and hormonal factors [100]. MiRNAs are a class of highly conserved, small (19–25 nucleotides) noncoding RNA molecules that mediate post-transcriptional gene expression regulation by inhibiting mRNA translation or promoting its degradation [101]. MiRNAs are key regulators of diverse cellular processes including proliferation, angiogenesis, differentiation, and apoptosis. Circulating miRNAs can be detected in bodily fluids, which makes them attractive targets as non-invasive diagnostic or prognostic biomarkers for different diseases [102]. MiRNAs have emerged as promising biomarkers reflecting specific mechanisms within different MINOCA etiologies. A distinct signature comprising increased miR-16 and miR-26a and decreased miR-1 and miR-133 was reported to characterize coronary microvascular spasm, while miR-145 and miR-222 were associated with epicardial spasm [103]. Further studies identified miR-155-5p, miR-483-5p, and miR-451a as biomarkers for plaque rupture [104]. In SCAD, a case–control study identified four upregulated miRNAs (let-7f-5p, miR-146a-5p, miR-151a-3p, and miR-223-5p) able to discriminate SCAD from atherothrombotic AMI with an AUC of 0.879 [105]. Elevated circulating TGF-β-related proteins such as interleukin-8 (IL-8), TGF-β1, transforming growth factor-beta receptor 1 (TGFBR1), ET-1, and matrix metalloproteinase-2 (MMP-2) have also been reported in SCAD [105,106,107]. The extracellular matrix protein fibrillin-1, essential for elastin fiber integrity, has been implicated in SCAD through its interactions with integrins, bone morphogenetic proteins, and TGF-β signaling [108,109]. Elevated plasma fibrillin-1 levels were observed in SCAD patients [107,110], further supporting an association between vascular connective tissue vulnerability and spontaneous dissection.

**Table 2:** Biomarker signatures of distinct MINOCA subtypes.

MINOCA Subtype	Key Biomarkers	Pathophysiological Insights	References
SCAD	Fibrillin-1, let-7f-5p, miR-146a-5p, miR-151a-3p, miR-223-5p, IL-8, TGF-β1, TGFBR1, ET-1, MMP-2	ECM instability, dysregulated TGF-β pathway	[100,105,106,107,110]
Atherosclerotic plaque disruption	sCD40L, NLR, WMR, NPR, miR-155-5p, miR-483-5p, miR-451a	Atherothrombosis	[80,97,104]
CAS	CRP/hs-CRP, ET-1, IL-6, suPAR, PAI-1, serotonin, cystatin-C, SELENBP1, VCL, miR-16, miR-26a, miR-1, miR-133, miR-145, miR-222	Endothelial dysfunction	[73,75,76,77,79,86,88,90,93,94,95,103]
CMD	CRP/hs-CRP, ET-1, IL-6, Lp(a), suPAR, PAI-1, serotonin, NPY, renalase, NLR, WMR, NPR	Microvascular inflammation	[65,73,74,78,79,80,84,85,86,87,88,89,90,91,92]
Embolic	Homocysteine, von Willebrand factor, D-dimer, protein C, protein S	Pro-thrombotic state	[2,21,72,98,99]

Abbreviations: CAS, coronary artery spasm; CMD, coronary microvascular dysfunction; CRP, C-reactive protein; ECM, extracellular matrix; ET-1, endothelin-1; hs-CRP, high-sensitivity C-reactive protein; IL-6, interleukin-6; IL-8, interleukin-8; Lp(a), lipoprotein(a); MMP-2, matrix metalloproteinase-2; NLR, neutrophil-to-lymphocyte ratio; NPR, neutrophil-to-platelet ratio; NPY, neuropeptide-Y; PAI-1, plasminogen activator inhibitor-1; SCAD, spontaneous coronary artery dissection; SELENBP1, selenium-binding protein 1; sCD40L, soluble CD40 ligand; suPAR, soluble urokinase-type plasminogen activator receptor; TGF-β, transforming growth factor-beta; TGFBR1, transforming growth factor-beta receptor 1; VCL, vinculin; WMR, white blood cell count-to-mean platelet volume ratio.

## 7. Prognostic Biomarkers in MINOCA

Although historically considered a benign condition, contemporary data demonstrate that patients with MINOCA remain at substantial risk of adverse CV outcomes [5,6]. Accordingly, accurate prognostic stratification is essential. Beyond traditional clinical predictors, several serum biomarkers have emerged as valuable tools for risk assessment in this heterogeneous population.

### 7.1. Prognostic Role of Myocardial Injury Biomarkers

Several studies have reported that MINOCA patients exhibit lower troponin levels than those with MI-CAD, suggesting less extensive myocardial damage in this cohort [27,28,29,30,31]. The prognostic value of hs-cTnT in MINOCA, compared with MI-CAD, was investigated in a large SWEDEHEART registry-based study [35]. Overall, hs-cTnT levels were markedly higher in MI-CAD than in MINOCA (618 [150–2346] vs. 180 [69–467] ng/L; *p* < 0.001). In both groups, hs-cTnT was significantly higher in patients who experienced CV mortality or MACE, but not all-cause mortality. In adjusted analyses, ln-transformed hs-cTnT levels in MINOCA patients independently predicted all-cause mortality [HR 1.32 (95% CI 1.11–1.56)], CV mortality [HR 2.11 (95% CI 1.51–2.96)] and MACE [HR 1.44 (95% CI 1.20–1.72)], based on Cox regression models in which proportional hazards assumptions were verified. Hs-cTnT (ln) also predicted readmissions for HF [HR 1.51 (95% CI 1.51–2.96)], but not non-fatal AMI or stroke. Interaction analyses suggested that hs-cTnT (ln) was at least as prognostic in MINOCA as in MI-CAD [35]. Similarly, in a cohort of 337 consecutive patients with a working diagnosis of MINOCA, patients were stratified according to the extent of high-sensitivity cardiac troponin I (hs-cTnI) elevation during hospitalization (≤5-times ULN, >5–≤20-times, and >20-times). Over a mean follow-up of 516 days, patients in the highest hs-cTnI group had significantly greater all-cause mortality and a borderline association with higher rates of major adverse cardiac and cerebrovascular events (MACCE). However, in multivariable Cox regression, hs-cTnI levels were no longer independently predictive of mortality or MACCE, suggesting that the initial unadjusted association may have been influenced by confounding clinical factors [36].

Taken together, these data suggest that although elevated cardiac troponin levels in MINOCA are associated with more severe myocardial injury and worse unadjusted outcomes, their independent prognostic value is less consistent and may be modulated by comorbidities and clinical presentation.

### 7.2. Prognostic Role of Inflammation Biomarkers

#### 7.2.1. C-Reactive Protein

In a large cohort of 9092 MINOCA patients, Nordenskjöld et al. showed that higher CRP levels predicted MACE in univariate analysis but lost significance after multivariable adjustment [HR 1.01 (95% CI 1.00–1.03); *p* = 0.241]. However, higher CRP remained independently associated with all-cause mortality in adjusted models [HR 1.03 (95% CI 1.01–1.06); *p* = 0.005] [49]. Other studies have reported stronger independent associations between CRP and adverse outcomes. In a large SWEDEHEART registry-based cohort, CRP levels were independently associated with both MACE and all-cause mortality in MINOCA patients [46], and similar findings were reported in a smaller cohort by Ciliberti et al., where admission CRP was a significant predictor of MACE [HR 1.47 (95% CI 1.06–2.07); *p* = 0.005] [50]. Overall, these findings suggest that while elevated CRP is frequently associated with worse outcomes in MINOCA, the strength and independence of this relationship vary across studies and analytical models.

#### 7.2.2. White Blood Cell Count

WBCc and its subtypes have long been studied as inflammatory biomarkers predicting CV outcomes in patients with CAD [111]. Recent evidence suggests that NLR, PLR, and NPR are useful markers of systemic inflammation and are associated with poor clinical outcomes in various CV diseases, including ACS [112,113,114]. In a retrospective cohort of 72 MINOCA patients and 248 controls with normal coronary angiograms, NLR at admission was significantly higher in the MINOCA group. During follow-up, 6 deaths occurred among MINOCA patients. Univariate Cox regression analysis identified NLR as a significant predictor of mortality [HR 1.24 (95% CI 1.09–1.41); *p* = 0.001]. By contrast, although PLR values were also higher in MINOCA patients, the difference did not reach statistical significance, likely due to limited sample size [115]. Systemic inflammation response index (SIRI) is a novel composite marker calculated from neutrophil, monocyte, and lymphocyte absolute counts [116] that has recently emerged as a reliable prognostic marker in ACS [117]. In a cohort of 259 MINOCA patients, those who developed MACE had significantly higher baseline SIRI than those without events. In multivariable Cox regression, high SIRI remained an independent predictor of adverse outcomes [HR 3.19 (95% CI 1.94–5.24); *p* < 0.001] [55]. The WMR is another simple inflammatory biomarker that has been shown to predict unfavorable outcomes in various CV conditions, including AMI [118]. WMR, as well as NPR, showed strong prognostic relevance in 274 MINOCA patients, with Kaplan–Meier curves demonstrating worse outcomes in the high-WMR (>701) and high-NPR groups (>0.03). Notably, the combination of high WMR and high NPR identified the subgroup with the highest long-term MACE incidence. Multivariable Cox regression confirmed that concomitant elevation of WMR and NPR independently predicted MACE, with the strongest hazard ratio [HR 2.51 (95% CI 1.27–4.96); *p* = 0.008] [80]. Another observational study of 335 MINOCA patients investigated several immune-inflammatory biomarkers: neutrophil-to-high-density lipoprotein cholesterol ratio (NHR), monocyte-to-high-density lipoprotein cholesterol ratio (MHR), lymphocyte-to-high-density lipoprotein cholesterol ratio (LHR), platelet-to-high-density lipoprotein cholesterol ratio (PHR), systemic immune-inflammation index (SII), SIRI, and aggregate index of systemic inflammation (AISI) [119]. Levels of NHR, MHR, LHR, PHR, SII, SIRI, and AISI were higher in patients who experienced MACE compared with those without events. Multiple logistic regression adjusted for confounders showed that NHR, MHR, PHR, SII, SIRI, and AISI were independently associated with MACE in MINOCA patients. The combination of these indices improved the predictive accuracy for MACE (AUC = 0.804) [119].

#### 7.2.3. Fibrinogen

Fibrinogen, a key component of coagulation pathway and acute-phase reactant, contributes to inflammation and atherosclerotic progression by promoting endothelial injury through the release of growth factors and smooth muscle proliferation [120,121]. Elevated serum fibrinogen level has been independently associated with CAD severity [121]. Conversely, low serum albumin has been linked to the no-reflow phenomenon [122], and hypoalbuminemia has been associated with increased risk of in-hospital cardiogenic shock, resuscitated cardiac arrest, and death [123]. Albumin deficiency impairs platelet inhibition and enhances inflammatory and oxidative injury [124]. The fibrinogen-to-albumin ratio (FAR), defined as the ratio of serum fibrinogen to serum albumin levels [125], has emerged as a novel inflammatory marker associated with adverse outcomes in CV disease, including AMI [120,121,122,123,124]. In a cohort of 1031 MINOCA patients, FAR was evaluated by stratifying individuals into high and low groups according to the median value. The high FAR group had more than twice the incidence of MACE compared with the low FAR group (21.2% vs. 9.3%; *p* < 0.001). In multivariable Cox regression, elevated FAR remained an independent predictor of long-term MACE after adjustment for conventional risk factors [HR 2.76 (95% CI 1.95–3.89); *p* < 0.001] [126].

### 7.3. Prognostic Role of Neurohormonal and Endocrine Biomarkers

#### 7.3.1. Natriuretic Peptides

Natriuretic peptides are well-established markers for diagnosing and assessing prognosis in patients with HF. NT-proBNP has been consistently associated with adverse events in patients with AMI [127,128,129]. In a prospective study including 59 consecutive patients with an initial angiographic diagnosis of MINOCA but without clinical signs of HF and with left ventricular ejection fraction (LVEF) ≥ 40%, 71.7% had NT-proBNP > 125 pg/mL, with a median value of 286 [100–698] pg/mL. NT-proBNP ≥125 pg/mL was significantly associated with post-discharge rehospitalization [54]. In a retrospective cohort of 259 MINOCA patients, those who developed MACE had significantly higher baseline NT-ProBNP levels than those without events. Kaplan–Meier analysis confirmed that elevated NT-ProBNP was associated with increased MACE risk, and multivariable Cox regression identified high NT-proBNP as an independent predictor of adverse outcomes [HR 2.245 (95% CI 1.432–3.519); *p* < 0.001] [55].

#### 7.3.2. Thyroid Hormones

There is a strong relationship between thyroid hormones and CV system [130]. Low free triiodothyronine (fT3) levels are associated with worse prognosis in AMI [131]. Abdu et al. evaluated the association between low fT3 and clinical outcomes in 218 MINOCA patients without prior thyroid disease. Patients were divided into a low fT3 group (fT3 < 3.5 pmol/L) and a normal fT3 group (3.5–6.5 pmol/L). At 2-year follow-up, MACE incidence was higher in the low fT3 group compared with the normal fT3 group (25.4% vs. 13.2%; *p* = 0.031). Multivariate logistic regression identified higher fT3 as independently associated with a lower risk of MACE [132]. Low triiodothyronine syndrome (LT3S) is a common finding in acute illness and occurs in 15–20% of AMI patients [133]. LT3S (fT3 < 2.36 pg/mL with normal thyroid-stimulating hormone) has been associated with a significantly higher MACE incidence among MINOCA patients and emerged as an independent predictor of MACE after multivariable adjustment in a Chinese observational study (HR 1.50 [95% CI 1.03–2.18]; *p* = 0.037) [134]. A reduced fT3/fT4 ratio is commonly observed in CV diseases, especially during acute illness such as ACS [135]. In an observational study of 1162 euthyroid MINOCA patients, lower fT3/fT4 tertiles were associated with a significantly higher incidence of MACE, and the risk of MACE increased progressively with decreasing fT3/fT4 tertiles, even after multivariable adjustment [136].

### 7.4. Prognostic Role of Cardiometabolic Biomarkers

#### 7.4.1. Acute Hyperglycemia and Chronic Glycemic Status

The prevalence of admission high glucose level (aHGL) in AMI ranges from 25 to 50% depending on the cut-off used [137,138], and approximately 10–20% of non-diabetic AMI patients present with significant admission hyperglycemia [139]. Whether elevated admission glucose reflects more extensive myocardial damage or acts as a prognostic marker in its own right remains debated [140]. Proposed mechanisms linking stress hyperglycemia to adverse outcomes include systemic immune activation, platelet dysfunction, altered thrombotic-fibrinolytic balance, autonomic imbalance, increased oxidative stress, endothelial dysfunction, and impaired myocardial contractility [141,142]. In a study comparing 2450 MI-CAD and 239 MINOCA patients, Paolisso et al. found that 16.7% of MINOCA patients had admission hyperglycemia. Hyperglycemic MI-CAD patients exhibited higher cardiac-troponin I (cTnI) levels, larger left ventricular end diastolic volumes, and lower LVEF than their normoglycemic counterparts, whereas MINOCA patients showed only trivial myocardial damage regardless of admission glucose [67]. The prognostic impact of aHGL was further evaluated in a study of 2431 patients classified according to the presence of aHGL (≥140 mg/dL) and AMI phenotype. aHGL was twice as frequent in MI-CAD as in MINOCA (37.6% vs. 16.3%) and was associated with a higher in-hospital arrhythmic burden in both groups, but with increased in-hospital mortality only in MI-CAD [143]. Because admission hyperglycemia may derive from chronic hyperglycemia or acute stress [144], the stress hyperglycemia ratio (SHR) was developed to better capture acute glycemic derangement, integrating admission glucose and glycated hemoglobin (HbA1c) [145]. Several clinical studies have shown that SHR is more strongly associated with in-hospital mortality and long-term MACE than admission glucose alone in AMI [144,146,147]. In a cohort of 410 MINOCA patients, Abdu et al. showed that higher SHR was independently associated with long-term MACE [148]. In a larger study, elevated SHR was independently associated with higher MACE risk [HR 2.30 (95% CI 1.21–4.38); *p* = 0.011]. Notably, SHR remained a robust predictor in patients with and without diabetes mellitus (DM), whereas admission glucose was no longer associated with MACE in diabetic patients [149]. HbA1c remains the gold standard for assessing long-term glycemic status [150]. In a prospective study, MINOCA patients were stratified into normoglycemia (HbA1c < 5.7%), prediabetes (5.7% ≤ HbA1c < 6.5%), and DM (HbA1c ≥ 6.5% or known DM). Patients with prediabetes and DM had significantly higher MACE incidence than the normoglycemic group (10.8%, 16.1%, 19.4%, respectively; *p* = 0.003). After multivariable adjustment, both prediabetes and DM remained significantly associated with increased MACE risk [151]. Insulin resistance, defined as reduced tissue responsiveness to insulin, significantly contributes to CV disease development and correlates with adverse outcomes [152,153]. Insulin resistance is central in type 2 DM pathogenesis but is also present in many non-diabetic individuals and frequently coexists with obesity, hypertension, and dyslipidemia [152]. The triglyceride-glucose index, derived from fasting TG and fasting blood glucose, has been proposed as a surrogate marker of insulin resistance [154]. Growing evidence suggests that triglyceride-glucose index is an independent predictor of adverse CV outcomes in ACS patients with or without DM [155,156]. MINOCA patients in higher triglyceride-glucose index tertiles experienced significantly more long-term MACE, and elevated triglyceride-glucose index remained independently associated with MACE after multivariable adjustment [HR 1.33 (95% CI 1.04–1.69); *p* = 0.02] [157].

#### 7.4.2. Atherogenic Lipoproteins and Residual Lipid Risk

Dyslipidemia remains a critical driver of CV risk. In a study of 196 patients, high LDL-C levels were identified as independent predictors of MACE in MINOCA [158]. Despite the well-established benefits of LDL-C lowering [159], residual risk persists even in statin-treated individuals with low LDL-C levels [160]. Interestingly, in the study by Nordenskjöld et al., lower TC levels were independently associated with MACE [HR 0.88 (95% CI 0.83–0.94); *p* < 0.001], all cause death [HR 0.83 (95% CI 0.77–0.91); *p* < 0.001], and HF, but not new AMI. When stratified according to statin use, only TC levels in patients without statins at admission but prescribed statins at discharge remained independently associated with MACE, likely reflecting different underlying pathophysiological mechanisms among MINOCA patients [49]. In patients with higher baseline cholesterol, initiation of statin therapy may substantially lower recurrent event risk, resulting in a paradoxical association between higher initial cholesterol and more favorable prognosis. Recent research has focused on the atherogenic role of TG, triglyceride-rich lipoproteins, and RC, which promote atherosclerotic CV disease [160]. RC (calculated as non-HDL-C minus LDL-C) represents the cholesterol content of triglyceride-rich lipoproteins, including chylomicron remnants, very-low-density lipoprotein, and intermediate-density lipoprotein [161]. RC has been identified as a residual risk factor in CAD [162], capable of accumulating in the subendothelial space and driving endothelial dysfunction, inflammation, and atherogenesis [163,164]. Gao et al. examined RC in a MINOCA cohort and found that the patients with higher median RC had a significantly higher incidence of MACE (16.9% vs. 11.5%; *p* = 0.009). High median RC remained an independent predictor of MACE in multivariable analysis [HR 1.41 (95% CI 1.03–1.93); *p* = 0.029] [165]. The atherogenic index of plasma (AIP), defined as the natural logarithm of the TG/HDL-C ratio, has emerged as a biomarker of lipid-related risk and a useful predictor of future CV events [166,167]. In an observational study of 421 MINOCA patients, AIP remained significantly associated with MACE both as a continuous and categorical variable [168].

Lp(a) is a low-density lipoprotein-like particle that plays an important role in atherothrombosis [169]. Elevated Lp(a) has been associated with increased risk of ischemic CV events [68,69,70]. Approximately 10–20% of the population has high Lp(a) levels, with wide inter-individual variability [71]. As Lp(a) concentrations are largely genetically determined and remain stable in adults, a once-in-a-lifetime measurement may be useful for risk stratification and prevention strategies. Although optimal cut-offs remain debated, thresholds of 40–60 mg/dL are commonly used and correlate well with vascular risk [71]. Elevated Lp(a) has been identified as an independent predictor of poor prognosis in CAD, suggesting a causal and continuous relationship between Lp(a) and CV risk [170,171]. The role of Lp(a) in MINOCA has only recently been explored, and data remain limited. Patients with higher Lp(a) levels exhibited significantly higher MACE incidence in a Chinese observational study, with MACE risk that increased with rising Lp(a) even after multivariable adjustment [172]. In another prospective multicenter cohort of 1042 AMI patients, including 76 MINOCA, high Lp(a) (>60 mg/dL) was an independent predictor of recurrent ischemic events or death during a median follow-up of 5.2 years [HR 1.44 (95% CI 1.02–2.03); *p* = 0.036] [45].

#### 7.4.3. Hyperuricemia

Hyperuricemia is increasingly recognized as a marker of poor CV prognosis in the general population [173] and in multiple CAD subgroups, including ACS [174]. Hyperuricemia is associated with increased intracellular oxidative stress, inflammation, vasoconstriction, and endothelial dysfunction [175,176]. Among MINOCA patients, hyperuricemia (≥420 μmol/L in men, ≥357 μmol/L in women) has been associated with higher MACE incidence and emerged as an independent predictor of MACE at multivariate analysis [177]. In the same study, rates of death, AMI, stroke, or revascularization did not differ significantly between groups; however, HF events were more frequent in the hyperuricemia group [177]. Another study of 249 MINOCA patients confirmed higher MACE incidence in the hyperuricemia group [178].

#### 7.4.4. Bilirubin

Serum total bilirubin (TB) has shown conflicting prognostic implications in CV disease. In stable populations, higher TB has been associated with lower CAD risk and complexity [179,180]. In contrast, in ACS, elevated TB has been linked to worse outcomes [181,182]. In a prospective cohort of 273 MINOCA patients, individuals were stratified according to an optimal TB cut-off of 0.9 mg/dL. High TB was present in 24.9% of patients and was associated with a significantly greater MACE incidence over 28 months (30.9% vs. 17.1%; *p* = 0.015). High TB remained an independent predictor of MACE both before (HR 1.90 [95% CI 1.12–3.22]; *p* = 0.018) and after multivariate adjustment (HR 2.04 [95% CI 1.05–3.94]; *p* = 0.034) [183].

### 7.5. Prognostic Role of Markers of Renal Dysfunction

Renal impairment is common in AMI and is as independent predictor of adverse events after coronary revascularization [184]. In the study by Nordenskjöld et al., higher creatinine levels were significantly associated with MACE in multivariable analysis [HR 1.01 (95% CI 1.00–1.03); *p* = 0.027]. Elevated creatinine was also an independent predictor of all-cause death [HR 1.02 (95% CI 1.00–1.03); *p* = 0.030], new AMI, and HF [49]. In a single-center study of 178 MINOCA patients followed for three years, nearly one-third (29.8%) had impaired kidney function [estimated glomerular filtration rate (eGFR) < 60 mL/min/1.73 m^2^]. This subgroup experienced more frequent in-hospital complications, including pulmonary edema, cardiogenic shock, cardiac arrest, and pneumonia. Renal dysfunction was strongly associated with worse outcomes: mortality was higher during hospitalization (7.55% vs. 0.8%), at one year (26.42% vs. 7.2%), and at three years (33.96% vs. 9.6%). Multivariate analysis confirmed older age, reduced eGFR, elevated creatinine, reduced LVEF, and ST-segment elevation as independent predictors of death. Notably, a substantial proportion of patients were eventually diagnosed with TTS [185]. In another study, 564 troponin-positive patients with non-obstructive coronary arteries (including myocarditis and TTS) were stratified by baseline renal function. Cumulative mortality increased progressively with worsening renal dysfunction, and each 10 mL/min/1.73 m^2^ decrement in eGFR was associated with an adjusted HR for mortality of 1.43 [(95% CI 1.20–1.72); *p* < 0.001] [186].

## 8. Future Implications

As diagnostic strategies for MINOCA advance, future clinical management is shifting toward precision medicine, with the goal of identifying the underlying ischemic mechanism and personalizing therapy. Current European and American guidelines emphasize structured diagnostic assessment—including early CMR, intracoronary functional testing, and multimodal biomarker evaluation—to clarify etiology and guide treatment pathways, though the implementation remains highly variable in practice [9,10]. The PROMISE trial (Precision Medicine in Myocardial Infarction with Non-Obstructive Coronary Arteries) is the first randomized controlled study specifically designed for MINOCA patients. In this trial, individuals with angiographically confirmed MINOCA were randomized 1:1 to receive either a precision medicine-guided management strategy or standard care. In the stratified treatment arm, patients underwent a comprehensive diagnostic protocol tailored to identify the underlying ischemic mechanism: OCT when plaque disruption was suspected on angiography; intracoronary acetylcholine provocation testing to assess for epicardial or microvascular spasm; transesophageal and/or contrast-enhanced echocardiography in cases where coronary embolism was considered; and CMR to characterize myocardial tissue abnormalities. Based on these findings, a mechanism-specific therapeutic regimen was initiated [187]. The recently published main results demonstrated that at 12-month follow-up, patients in the precision medicine group achieved significantly better angina-related quality of life, as captured by the Seattle Angina Questionnaire summary score. The incidence of the key secondary endpoint, a composite of all-cause mortality, AMI, stroke, HF hospitalization, and repeat coronary angiography, was numerically lower in the stratified group (2.2%) compared with standard care (8.5%), although not reaching statistical significance [188]. Importantly, the original study design also included blood sampling for circulating biomarkers and miRNAs profiling at the time of coronary angiography or within 12 h, with exploratory correlation of biomarker signatures (including ET-1, NPY, CRP, sCD40L, miR-16, miR-26a, miR-145, miR-222, miR-155-5p, miR-483-5p, and miR-451) with underlying mechanisms of MINOCA [187]. Although this sub-analysis has not yet been reported, it represents a promising step toward biomarker-based phenotyping in MINOCA.

In conclusion, future research is expected to build upon the available evidence by incorporating:

- Multi-omics biomarker platforms, combining proteomics, lipidomics, and circulating miRNAs profiling, to classify MINOCA mechanisms in real time;
- Integration of biomarker panels with advanced imaging and artificial intelligence-driven analytics to improve diagnostic accuracy and reduce delays in targeting specific etiologies;
- Sex-specific biomarker signatures to address mechanistic differences, particularly in SCAD and CMD, which disproportionately affect women;
- Biomarker-guided risk stratification, using biomarker levels to tailor the type and intensity of therapy based on predicted patient risk and underlying disease mechanisms.

## 9. Conclusions

Serum biomarkers offer a powerful opportunity to improve the understanding of MINOCA pathophysiology and may contribute to diagnostic evaluation and risk stratification during both the acute phase and long-term follow-up. Biomarkers reflecting myocardial injury, inflammation, endothelial dysfunction, neurohormonal activation, and metabolic stress provide complementary information on the heterogeneous mechanisms underlying MINOCA and its clinical outcomes. However, single biomarkers lack sufficient specificity, and current evidence supports their use mainly as adjunctive tools within a broader diagnostic framework that includes clinical assessment and multidimensional imaging. Emerging molecular biomarkers, including circulating miRNAs, and combined multi-marker strategies may further refine etiological classification in the future. Nevertheless, these approaches remain investigational, and prospective studies are needed to determine their reproducibility, clinical utility, and potential role in guiding management strategies in MINOCA.

## Figures and Tables

**Table 1:** Comparative serum biomarker profiles in acute myocardial injury syndromes.

Key Biomarkers	MINOCA	MI-CAD	TTS	Myocarditis	Differential Diagnostic Clues	Clinical Applicability	References
cTnT/cTnI/hs-cTnT/hs-cTnI	↑	↑↑	↑	↑/↑↑	In case of troponin disproportionately low vs. symptom severity consider TTS/myocarditis.	Clinical use	[25,27,28,29,30,31,32,33,34,35,36,37,38,39,40,41,42]
CK-MB	↑	↑↑	↑	↑/↑↑	Adds little beyond high-sensitivity troponin in contemporary practice.	Clinical use	[28,41,42]
CRP/hs-CRP	↑	↑/↑↑	↑	↑↑	A very high CRP relative to troponin strongly favors an inflammatory myocardial process (such as myocarditis) over ischemic necrosis.	Clinical use	[25,28,29,34,37,38,39,40,43,44,45,46,47,48,49,50,51,52,53]
GDF-15	↑/↑↑	↑	-	-	Higher in MINOCA than in MI-CAD at baseline but with converging values at follow-up.	Research use	[34]
IL-6	↑	↑↑	-	-	-	Research use	[47]
NT-proBNP and BNP	↑	↑/↑↑	↑↑	↑/↑↑	An elevated NT-proBNP or BNP to troponin ratio suggests TTS.	Clinical use	[25,37,38,39,40,41,42,43,44,54,55]
TG	↔	↑	↔	-	-	Clinical use	[43]
LDL-C	↔	↑	↔	-	-	Clinical use	[43]
HDL-C	↑	↔	↑	-	-	Clinical use	[43]
RC	↑	↔	-	-	-	Research use	[56]
MHR	↑	↔	-	-	-	Research use	[56]
Lp(a)	↔	↑	-	-	-	Clinical use	[45]
Plasminogen	↓	↔	-	-	-	Research use	[31]
Homocysteine	↑	↔	-	-	-	Research use	[31]
miR-16, miR-26a, let-7f	-	↔	↑	-	Higher in TTS than in STEMI.	Research use	[57]
miR-133a	-	↑	↔	-	Higher in STEMI than in TTS.	Research use	[57]
miR-125a-5p	-	↔	↓	-	miR-125a-5p is a known negative regulator of ET-1.	Research use	[57]
ET-1	-	↔	↑	-		Research use	[57]

**Symbols:** ↑ mild increase; ↑↑ moderate increase; ↓ decrease; ↔ stable or no significant change; - data not available. **Abbreviations:** BNP = B-type natriuretic peptide; CK-MB = creatine kinase-myocardial band; CRP = C-reactive protein; cTnI = cardiac troponin I; cTnT = cardiac troponin T; ET-1 = endothelin-1; GDF-15 = growth differentiation factor 15; HDL-C = high-density lipoprotein cholesterol; hs-CRP = high-sensitivity C-reactive protein; hs-cTnI = high-sensitivity cardiac troponin I; hs-cTnT = high-sensitivity cardiac troponin T; IL-6 = interleukin-6; LDL-C = low-density lipoprotein cholesterol; Lp(a) = lipoprotein(a); MHR = monocyte-to-high-density lipoprotein cholesterol ratio; MI-CAD = myocardial infarction with obstructive coronary artery disease; MINOCA = myocardial infarction with non-obstructive coronary arteries; NT-proBNP = N-terminal pro-B-type natriuretic peptide; RC = remnant cholesterol; TG = triglycerides; TTS = Takotsubo syndrome. Biomarkers are categorized according to their current clinical applicability (clinical vs. research use), based on the authors’ consensus informed by the available evidence. As the available evidence is predominantly observational and no randomized controlled trials are available, no formal grading of evidence strength was performed.

## Data Availability

No new data were created or analyzed in this study.

## Abbreviations

The following abbreviations are used in this manuscript:

ACS	Acute coronary syndrome
ADMA	Asymmetric dimethylarginine
AGRP	Agouti-related protein
aHGL	Admission high glucose level
AIP	Atherogenic index of plasma
AISI	Aggregate index of systemic inflammation
AMI	Acute myocardial infarction
AUC	Area under the curve
BNP	B-type natriuretic peptide
CAD	Coronary artery disease
CAS	Coronary artery spasm
CCL-15	C-C motif chemokine ligand 15
CCL-21	C-C motif chemokine ligand 21
CCL-8	C-C motif chemokine ligand 8
CFR	Coronary flow reserve
CK-MB	Creatine kinase–myocardial band
CMD	Coronary microvascular dysfunction
CMR	Cardiac magnetic resonance
CRP	C-reactive protein
cTnI	Cardiac troponin I
cTnT	Cardiac troponin T
CV	Cardiovascular
CXCL-1	C-X-C motif chemokine ligand 1
CXCL-6	C-X-C motif chemokine ligand 6
DM	Diabetes mellitus
EAT	Epicardial adipose tissue
ECG	Electrocardiogram
eGFR	Estimated glomerular filtration rate
EMB	Endomyocardial biopsy
ET-1	Endothelin-1
FAR	Fibrinogen-to-albumin ratio
fT3	Free triiodothyronine
fT4	Free thyroxine
GDF-15	Growth differentiation factor 15
HbA1c	Glycated hemoglobin
HDL-C	High-density lipoprotein cholesterol
HF	Heart failure
HR	Hazard ratio
hs-CRP	High-sensitivity C-reactive protein
hs-cTnI	High-sensitivity cardiac troponin I
hs-cTnT	High-sensitivity cardiac troponin T
IL-1Ra	Interleukin-1 receptor antagonist protein
IL-20	Interleukin-20
IL-6	Interleukin-6
IL-8	Interleukin-8
IMR	Index of microcirculatory resistance
IVUS	Intravascular ultrasound
LDL-C	Low-density lipoprotein cholesterol
LHR	Lymphocyte-to-high-density lipoprotein cholesterol ratio
Lp(a)	Lipoprotein(a)
LT3S	Low triiodothyronine syndrome
LVEF	Left ventricular ejection fraction
MACCE	Major adverse cardiac and cerebrovascular events
MACE	Major adverse cardiovascular events
MHR	Monocyte-to-high-density lipoprotein cholesterol ratio
MI-CAD	Myocardial infarction with obstructive coronary artery disease
MINOCA	Myocardial infarction with non-obstructive coronary arteries
miRNAs	microRNAs
MMP-2	Matrix metalloproteinase-2
MPO	Myeloperoxidase
NEMO	Nuclear factor-κB essential modulator
NHR	Neutrophil-to-high-density lipoprotein cholesterol ratio
NLR	Neutrophil-to-lymphocyte ratio
NPR	Neutrophil-to-platelet ratio
NPY	Neuropeptide-Y
NSTEMI	Non-ST-elevation myocardial infarction
NT-proBNP	N-terminal pro-B-type natriuretic peptide
OCT	Optical coherence tomography
OR	Odds ratio
OSM	Oncostatin M
PAI-1	Plasminogen activator inhibitor-1
PAPPA	Pappalysin-1
PCAT	Pericoronary adipose tissue
PHR	Platelet-to-high-density lipoprotein cholesterol ratio
PlGF	Placental growth factor
PLR	Platelet-to-lymphocyte ratio
PSGL-1	P-selectin glycoprotein ligand-1
RC	Remnant cholesterol
REN	Renin
SCAD	Spontaneous coronary artery dissection
sCD40L	Soluble CD40-ligand
SELENBP1	Selenium binding protein-1
SHR	Stress hyperglycemia ratio
SII	Systemic immune-inflammation index
SIRI	Systemic inflammation response index
STEMI	ST-elevation myocardial infarction
suPAR	Soluble urokinase plasminogen activator surface receptor
sVCAM-1	Soluble vascular cell adhesion molecule-1
TB	Total bilirubin
TC	Total cholesterol
TEE	Transesophageal echocardiography
TG	Triglycerides
TGF-β	Transforming growth factor-β
TGFBR1	Transforming growth factor-beta receptor 1
tPA	Tissue-type plasminogen activator
TRANCE	TNF-related activation-induced cytokine
TTS	Takotsubo syndrome
ULN	Upper limit of normal
VCL	Vinculin
WBCc	White blood cell count
WMR	White blood cell count-to-mean platelet volume ratio
